# Supplementary material for: Similarity and quality metrics for MR image-to-image translation
Source: Sci Rep. 2025 Jan 31;15:3853. doi: 10.1038/s41598-025-87358-0 (PMC11785996; doi:10.1038/s41598-025-87358-0)
Supplement: Supplementary file 1 — Supplementary Information. [file 41598_2025_87358_MOESM1_ESM.pdf]

Similarity and Quality Metrics for MR Image-to-Image Translation (Supplement)

Melanie Dohmen, Mark A. Klemens, Ivo M. Baltruschat, Tuan Truong, Matthias Lenga

A Supplementary Methods

A.1 Notation

Let I be an image with intensity $I(\mathbf{x})$ at pixel location $\mathbf{x}$. A three-dimensional image $I \in \mathbb{R}^{w\times h\times d}$ of height h, width w and depth d consists of $|I| = w \cdot h \cdot d$ pixels, with pixel locations denoted as $\mathbf{x} = (x_1, x_2, x_3) \in \mathbb{N}^3$ with $0 \le x_1 < h$, $0 \le x_2 < w$, $0 \le x_3 < d$. Accordingly, a two-dimensional image is defined as $I \in \mathbb{R}^{h\times w}$ with pixel locations $\mathbf{x} = (x_1, x_2)$ and $|I| = h \cdot w$ pixels in total. Let $I_{\max}$ denote the maximum intensity, $I_{\min}$ the minimum intensity, μ_I the mean intensity, σ_I the standard deviation of all image intensities in I. The k-th percentile $I_{k\%} \in \mathbb{R}$ of the image I is the smallest intensity value, such that a fraction of $k\%$ of all pixels in I have lower or equal intensity value. The median of all image intensities in I is $I_{50\%}$. The interquartile range (IQR) is $I_{75\%} - I_{25\%}$.

A.2 Calculation of Normalization Methods

The calculation for all normalization methods is found here, the notation is detailed in Sec. A.1.

Minmax

Given a (sub-) intensity range $[i_1, i_2]$ the image intensities are shifted and scaled to meet a target intensity range $[j_1, j_2]$. For the default choice of $i_1 = I_{\min}$, $i_2 = I_{\max}$, $j_1 = 0$ and $j_2 = 1$, these parameters are dropped.

$$\mathrm{Minmax}_{[i_1,i_2]\mapsto[j_1,j_2]}(I) = \frac{I - i_1}{i_2 - i_1} \cdot (j_2 - j_1) + j_1 \qquad \mathrm{Minmax}(I) = \frac{I - I_{\min}}{I_{\max} - I_{\min}} \tag{1}$$

In case of constant images with $I_{\min} = I_{\max}$, the division by zero is omitted, such that the result is a constant image $\mathrm{Minmax}(I) = j_1$.

cMinmax

Clipped Minmax normalization is equal to Minmax normalization with previous clipping at the percentiles $I_{p\%}$ and $I_{q\%}$. If only $p < 50\%$ is given, the default is $q = 100\% - p = 95$ and $j_1 = 0$ and $j_2 = 1$, which reduces the notation.

$$\mathrm{cMinmax}_{[I_{p\%},I_{q\%}]\mapsto[j_1,j_2]}(I) = \mathrm{Minmax}_{[I_{p\%},I_{q\%}]\mapsto[j_1,j_2]}((\mathrm{clip}_{I_{p\%},I_{q\%}}(I)) \qquad \mathrm{clip}_{[I_{p\%},I_{q\%}]}(I) = \begin{cases} I_{p\%} & \text{, if } I(\mathbf{x}) \le I_{p\%} \\ I_{q\%} & \text{, if } I(\mathbf{x}) \ge I_{q\%} \\ I(\mathbf{x}) & \text{, else} \end{cases} \tag{2}$$

$$\mathrm{cMinmax}_{p\%}(I) = \mathrm{Minmax}(\mathrm{clip}_{[I_{p\%},I_{100\%-p\%}]}(I))$$

In case of (nearly) constant images with $I_{p\%} = I_{q\%}$, the result becomes a constant image with $\mathrm{cMinmax}(I) = j_1$.

Zscore

$$\mathrm{Zscore}(I) = \frac{I - \mu_I}{\sigma_I} \tag{3}$$

In case of constant images with $\sigma_I = 0$, the division by zero is omitted, such that $\mathrm{ZScore}(I) = 0$.

Quantile

$$\mathrm{Quantile}(I) = \frac{I - I_{50\%}}{I_{75\%} - I_{25\%}} \tag{4}$$

In case of a large fraction of equal image values, i.e. $I_{25\%} = I_{75\%}$, the division by zero is omitted, such that $\mathrm{Quantile}(I) = I - I_{50\%}$.

Binning

The image intensities are mapped to B bin values.

$$\mathrm{Bin}_B(I) = \min(B-1, \lfloor \mathrm{Minmax}_{[I_{\min},I_{\max}]\mapsto[0,B-1]} \rfloor) = \min(B-1, \lfloor B \cdot (I - I_{\min})/(I_{\max} - I_{\min}) \rfloor) \tag{5}$$

A.3 Metric Calculation

Reference and non-reference metrics are ordered alphabetically. The notation is detailed in Sec. A.1.

BE (Blur-Effect)

For each dimension $d = 1, ..., n$ of I, a blurred version $\tilde{I}$ of image I is created by convolution with a uniform kernel $U_{k,d}$ of size k along d. The absolute differences of neighboring pixels along d are in $\tilde{I}$ and I as $\tilde{D}$ and D respectively. D_d and $\tilde{D}_d$ can be seen as the gradients or edge images of the original image I and its blurred version $\tilde{I}$. Then the sum of positive differences $D - \tilde{D}$ is related to the sum of differences D only as a measure of blurriness. BE is implemented in the scikit-image python library [1] with a default of $k = 11$. In detail, we compute for all d:

$$\begin{aligned}
&\tilde{I}_d = \mathrm{conv}(U_{k,d}, I) && \\
&D_d = |\nabla_d(I)| && S_d = \sum_{\mathbf{x}} D_d(\mathbf{x}) \\
&\tilde{D}_d = |\nabla_d(\tilde{I}_d)| && \tilde{S}_d = \sum_{\mathbf{x}} \max(0, D_d(\mathbf{x}) - \tilde{D}_d(\mathbf{x}))
\end{aligned} \tag{6}$$

where $\nabla_d(I)$ denotes the differential of image I in dimension d and $\mathbf{x}$ denotes all pixel locations in D_d. The final Blur-Effect is defined as

$$\mathrm{BE} = \max_d \frac{S_d - \tilde{S}_d}{S_d} \tag{7}$$

BEW (Blurred Edge Widths)

First, edge pixels are detected along all dimensions d as $E_d(\mathbf{x}) = 1$ and non-edge pixels as $E_d(\mathbf{x}) = 0$, e.g. with the canny algorithm [2]. Second, edge pixels are traced along their detected dimension to find the next pixel with a differently signed gradient, which marks the end of the edge. The summed distances of the edge pixel to the ends of the edge determine the edge width $W_d(\mathbf{x})$ for dimension d.

Let d be a number of dimensions $\in 0, 1, ..., n$, then the BEW metric is defined as:

$$\mathrm{BEW}(\mathbf{x}) = \frac{1}{n} \cdot \sum_d \frac{\sum_{\mathbf{x}} W_d(\mathbf{x})}{\sum_{\mathbf{x}} E_d(\mathbf{x})} \tag{8}$$

BR (Blur Ratio)

In order to identify edge pixels, the image gradient along dimension d and the mean absolute value μ_{D_d} are computed:

$$D_d = |\nabla_d(I)| \qquad \mu_{D_d} = \frac{1}{|I|} \sum_{\mathbf{x}} D_d(\mathbf{x}) \tag{9}$$

With two further criteria, a binary map of edge pixels $E_d(\mathbf{x}) \in \{0, 1\}$ is derived from D_d. First, all pixels with gradient values lower or equal to the mean gradient are set to 0 in the edge candidate gradient map C_d. Second, the gradient value must exceed the values of its direct neighbors in dimension d:

$$C_d(\mathbf{x}) = \begin{cases} D_d(\mathbf{x}) & \text{, if } D_d(\mathbf{x}) > \mu_{D_d} \\ 0 & \text{, otherwise} \end{cases} \qquad E_d(\mathbf{x}) = \begin{cases} 1 & \text{, if } C_d(\mathbf{x}) > C_d(\mathbf{x}_1, ..., \mathbf{x}_d + 1, ..., \mathbf{x}_n) \\ & \quad \text{and } C_d(\mathbf{x}) > C_d(\mathbf{x}_1, ..., \mathbf{x}_d - 1, ..., \mathbf{x}_n) \\ 0 & \text{, otherwise} \end{cases} \tag{10}$$

In order to determine blurred pixels, the average intensity $A_d(\mathbf{x})$ of the neighbors of $\mathbf{x}$ in dimension d is defined.

$$A_d(\mathbf{x}) = \frac{|I(x_1, ..., x_d - 1, ..., x_n) - I(x_1, ..., x_d + 1, ..., x_n)|}{2} \tag{11}$$

And then related to the pixel intensity to define the inverse blurriness IB_d for dimension d. A threshold t_{IB} identifies blurred pixels, where the inverse blurriness of all dimensions d falls below t_{BR}.

$$\begin{aligned}
&IB_d(\mathbf{x}) \frac{|I(\mathbf{x}) - A_d(\mathbf{x})|}{A_d(\mathbf{x})} && B(\mathbf{x}) = \begin{cases} 1 & \text{, if } \max_d(IB_d(\mathbf{x})) < t_{\mathrm{IB}} \\ 0 & \text{, otherwise} \end{cases} \\
&\mathrm{BR} = \frac{\sum_{\mathbf{x}} B(\mathbf{x})}{\sum_{\mathbf{x}} \max_d(E_d(\mathbf{x}))} && \mathrm{MB} = \frac{\sum_{\mathbf{x}} \max_d IB_d(\mathbf{x})}{\sum_{\mathbf{x}} B(\mathbf{x})}
\end{aligned} \tag{12}$$

Based on experiments, the original paper proposes a threshold of $t_{\mathrm{IB}} = 0.1$. The BR metric is defined as the ratio of the total number of blurred pixels and the total number of edge pixels

BRISQUE (Blind/Reference-less Image Spatial Quality Evaluator)

As a first step, the image I is normalized with a modified zscore method[3] including a stabilizing constant $C = 1$ to I', also called mean subtracted contrast normalized (MSCN) coefficients.

$$I' = \frac{I - \hat{\mu}_I}{\hat{\sigma}_I + C} \tag{13}$$

In a second step, pairwise products of image intensities in a direct neighborhood are calculated:

$$\begin{aligned} H(I',\mathbf{x}) &= I'(\mathbf{x}) \cdot I'(x_1+1, x_2) && \text{(horizontal)} \\ V(I',\mathbf{x}) &= I'(\mathbf{x}) \cdot I'(x_1, x_2+1) && \text{(vertical)} \\ D_1(I',\mathbf{x}) &= I'(\mathbf{x}) \cdot I'(x_1+1, x_2+1) && \text{(first diagonal)} \\ D_2(I',\mathbf{x}) &= I'(\mathbf{x}) \cdot I'(x_1-1, x_2+1) && \text{(second diagonal)} \end{aligned} \tag{14}$$

Third, a general Gaussian distribution is fitted to I' with parameters shape s and variance v as well as asymmetric Gaussian distributions are fitted to each of H, V, D_1 and D_2 with parameters shape s, mean m, left variance lv, and right variance rv, yielding $1 \cdot 2 + 4 \cdot 4 = 18$ parameters. Finally, the BRISQUE quality score is predicted from these features by a trained support vector regressor.

CW-SSIM (Complex-Wavelet Structural Similarity Index Measure

In the complex wavelet transform domain, suppose $\mathbf{c}_{\mathbf{x},i} = \{c_{\mathbf{x},i} | i = 1,_N\}$ and $\mathbf{d}_{\mathbf{x},i} = \{d_{\mathbf{x},i} | i = 1,_N\}$ are two sets of coefficients extracted at the same spatial location $\mathbf{x}$ in the same wavelet subbands of the two images I and R being compared, respectively. Then, CW-SSIM is defined as

$$\mathrm{CW-SSIM}(I,R) = \frac{1}{|I|} \sum_{\mathbf{x} \in R} \frac{2|\sum_{i=1}^{N} c_{\mathbf{x},i} \cdot d^*_{\mathbf{x},i}| + K}{\sum_{i=1}^{N} |c_{\mathbf{x},i}|^2 + \sum_{i=1}^{N} |d_{\mathbf{x},i}|^2 + K} \tag{15}$$

where $d^*_{\mathbf{x},i}$ is the complex conjugate of $d_{\mathbf{x},i}$ and K is a constant to improve robustness, where the local signal-to-noise ratios are low. For our experiments, we used an implementation[4] with $K = 1 \cdot 10^{-12}$ and images were Minmax normalized to range $(0, 255)$.

CPBD (Cumulative Probability of Blur Detection)

The probability of detecting blur in a pixel $\mathbf{x}$ of an edge block e can be expressed as an exponential function of $p(\mathbf{x}, e)$. Let $\mathscr{X}$ be the set of processed edge pixels, i.e. the set of detected edge pixels located in an edge block $e \in \mathscr{E}$. Then the CPBD metric corresponds to the cumulative probability of detecting blur in any of the processed edge pixels with a probability ≤ 0.63.

$$p(\mathbf{x}, e) = 1 - \exp\left(-\left|\frac{W_0(\mathbf{x})}{\mathrm{jnb}(e,L)}\right|\right)^{\beta} \qquad \mathscr{X} := \left\{\mathbf{x} \mid \max_d E_d(\mathbf{x}) = 1 \wedge \exists e \in \mathscr{E} : \mathbf{x} \in e\right\} \tag{16}$$

$$\mathrm{CPBD}(I) = \sum_{\mathbf{x} \in \mathscr{X}} \begin{cases} 1/|\mathscr{X}| & \text{, if } p(\mathbf{x},e) \leq 0.63 \\ 0 & \text{, else} \end{cases} \tag{17}$$

We employed canny edge detection with a lower threshold at $0.1 \cdot L$ and a higher threshold of $0.2 \cdot L$, and scaled $\mathrm{jnb}(e)$ with L as defined in Eq. (21).

DSC (Dice Similarity Coefficient)

For segmentations S_I and S_R of images I and R respectively, the DSC relates the intersection of both segmentations to the sum of their sizes.

$$\mathrm{DSC}(S_I, S_R) = \frac{2|S_I \cap R_I| + \varepsilon}{|S_I| + |R_I| + \varepsilon} \tag{18}$$

The small constant $\varepsilon > 0$ is typically introduced, to assure that the DSC is not undefined in case both segmentations are empty.

DISTS (Deep Image Structure and Texture Similarity)

Given the feature maps $F_l^i(I)$ and $F_l^i(R)$ from layer l and channel i of a pre-trained network, the structure similarity S_l^i and the texture similarity T_l^i between the feature maps were defined as

$$S_l^i(I,R) = \frac{2\,\mu_{F_l^i(I)} \cdot \mu_{F_l^i(R)} + C_1}{\mu^2_{F_l^i(I)} + \mu^2_{F_l^i(R)} + C_1} \qquad T_l^i(I,R) = \frac{2\,\sigma_{F_l^i(I)} \cdot \sigma_{F_l^i(R)} + C_2}{\sigma^2_{F_l^i(I)} + \sigma^2_{F_l^i(R)} + C_2} \tag{19}$$

where C_1 and C_2 are positive constants. The overall DISTS metric is obtained by averaging weighted combinations of structure and texture similarities across network layers at different depths levels

$$\mathrm{DISTS}(I,R) = \sqrt{\sum_l \sum_i \left(\alpha_l^i\, S_l^i(I,R) + \beta_l^i\, T_l^i(I,R)\right)^2} \tag{20}$$

where α_l^i, β_l^i are optimized weighting factors for each channel i and layer l.

JNB (Just Noticeable Blur)

The idea of blurred edge widths (BEW) is extended with a notion of just noticeable blur. The image I is evaluated in smaller blocks b of size s_b. Only blocks $e \in \mathscr{E}$ with a sufficient fraction ($T > 0.002$) of edge pixels are considered. The edge widths are additionally weighted with the just noticeable blur width $\mathrm{jnb}(e,L)$ depending on the maximal intensity range per block e and the data range L, which is 255 by default for 8-bit images. The exponential parameter $\beta = 3.6$ was also used for adjustments.

$$\mathscr{E} = \left\{ b \;\middle|\; \frac{\sum_{\mathbf{x}\in b} \max_d E_d(\mathbf{x})}{s_b} > 0.002 \right\} \qquad \mathrm{jnb}(e,L) = \begin{cases} 5 & \text{, if } \frac{e_{\max}-e_{\min}}{L} <= \frac{50}{255} \\ 3 & \text{, else} \end{cases} \tag{21}$$

$$\mathrm{JNB}(I) = \frac{1}{|\mathscr{E}|} \cdot \sum_{e\in\mathscr{E}} \left(\sum_{\mathbf{x}\in e} \left(\frac{W_0(\mathbf{x})}{\mathrm{jnb}(e,L)} \right)^{\beta} \right)^{1/\beta} \tag{22}$$

LPIPS (Learned Perceptual Image Patch Similarity)

The LPIPS metric extracts feature maps $F_l^i(I)$ and $F_l^i(R)$ from layer l and channel i of the pre-trained network. The feature maps are unit-scaled to $\hat{F}_l^i(I), \hat{F}_l^i(R)$, weighted channel-wise with vectors w_l and subtracted. Then the corresponding L_2 norms are averaged spatially and summed to obtain the LPIPS distance $\mathrm{LPIPS}(I,R)$:

$$\mathrm{LPIPS}(I,R) = \sum_l \frac{1}{|F_l|} \sum_{\mathbf{x}_l} \left| w_l \odot (\hat{F}_l(I) - \hat{F}_l(R))(\mathbf{x}_l) \right|_2^2 \tag{23}$$

where $\mathbf{x}_l$ denotes a pixel location in feature map F_l, $\odot$ denotes the channel-wise multiplication and $|\cdot|_2$ denotes the euclidean norm. The linear weights w_l^i were optimized with the Berkeley-Adobe Perceptual Patch Similarity (BAPPS) Dataset [5].

MAE (Mean Absolute Error)

$$\mathrm{MAE}(I,R) = \frac{1}{|I|} \sum_{\mathbf{x}} |R(\mathbf{x}) - I(\mathbf{x})| \tag{24}$$

MB (Mean Blur)

See the BR metric for details on how to compute the blurred edges B and the inverse blurriness IB. The MB metric is defined as the ratio of the summed inverse blurriness to the number of blurred pixels:

$$\mathrm{MB} = \frac{\sum_{\mathbf{x}} \max_d IB_d(\mathbf{x})}{\sum_{\mathbf{x}} B(\mathbf{x})} \tag{25}$$

MLC (Mean Line Correlation)

The mean line correlation (MLC) metric is defined as the mean correlation between directly neighbored image lines:

$$\mathrm{MLC} = \frac{1}{w} \sum_{x=1}^{w} \mathrm{PCC}\left(I_c(x), I_c(x+1)\right) + \frac{1}{h} \sum_{y=1}^{h} \mathrm{PCC}\left(I_r(y), I_r(y+1)\right) \tag{26}$$

MSE (Mean Squared Error)

$$\mathrm{MSE}(I,R) = \frac{1}{|I|}\sum_{\mathbf{x}}(R(\mathbf{x}) - I(\mathbf{x}))^2 \tag{27}$$

MSLC (Mean Shifted Line Correlation)

The mean shifted line correlation (MSLC) metric is defined as the mean correlation between image lines, that are separated by $\lfloor w/2 \rfloor$ or $\lfloor h/2 \rfloor$ respectively.

$$\mathrm{MLC} = \frac{1}{\lfloor w/2 \rfloor}\sum_{x=1}^{\lfloor w/2 \rfloor}\mathrm{PCC}\left(I_c(x), I_c(x+1)\right) + \frac{1}{\lfloor h/2 \rfloor}\sum_{y=1}^{\lfloor h/2 \rfloor}\mathrm{PCC}\left(I_r(y), I_r(y+1)\right) \tag{28}$$

MS-SSIM (Multi-Scale Structural Similarity Index Measure)

As for SSIM, luminance, contrast and structure are defined locally. For MS-SSIM they are calculated on different scales. Let I_M be the M-times low-pass filtered and downscaled version of image I. Then MS-SSIM is calculated locally as

$$\mathrm{local-MS-SSIM}(I,R,\mathbf{x}) = l(I_M,R_M,\mathbf{x})^{\alpha_M}\cdot\prod_{j=1}^{M}c(I_j,R_j,\mathbf{x})^{\beta_j}\cdot s(I_j,R_j,\mathbf{x})^{\gamma_j} \tag{29}$$

where the weighting factors $\alpha_j = \beta_j = \gamma_j$ were determined experimentally for $j = 1\ldots5$ as $[0.0448, 0.2856, 0.3001, 0.2363, 0.1333]$. Then the overall MS-SSIM value is averaged over all pixel locations:

$$\mathrm{MS-SSIM}(I,R) = \frac{1}{|I|}\sum_{\mathbf{x}\in R}l(I_M,R_M,\mathbf{x})^{\alpha_M}\cdot\prod_{j=1}^{M}c(I_j,R_j,\mathbf{x})^{\beta_j}\cdot s(I_j,R_j,\mathbf{x})^{\gamma_j} \tag{30}$$

MTV (Mean Total Variation)

$$\mathrm{MTV}(I) = \frac{1}{|I|}\sum_{\mathbf{x}}\sqrt{\sum_{d}\left(I(\mathbf{x}) - I(x_1,...,x_d+1,..,x_n)\right)^2} \tag{31}$$

NIQE (Natural Image Quality Evaluator)

The same 18 features are extracted as in the calculation of the BRISQUE metric. Additionally, The features are collected again on the image downscaled by factor two to obtain 36 features in total. This is done for all patches of size 96×96 in the image to be tested, and it was previously done for patches of reference training set consisting of undistorted natural images. The distribution of these features $f_1,\ldots,f_{36}$ can be fitted to a multivariate Gaussian model with mean ν and covariance matrix Σ. From patches of the reference dataset, ν_R and Σ_R were obtained. For any test image I, the fitted model parameters are denoted ν_I and Σ_I. The NIQE score is then defined as the difference between the fitted model parameters:

$$\mathrm{NIQE}(I) = \sqrt{\left((\nu_R-\nu)^{-T}\cdot(\frac{\Sigma_R-\Sigma_I}{2})^{-1}\cdot(\nu_R-\nu_I)\right)} \tag{32}$$

NMI (Normalized Mutual Information)

The joint probability distribution $p(I=i,R=r)$ represents the likelihood of pairs of intensity values $i = I(\mathbf{x})$ and $r = R(\mathbf{x})$ occurring at any pixel location $\mathbf{x}$ in I and R. The joint probability distribution is computed by counting how many pixel locations x in I and R and dividing this number by the number of pixels $|I|$. Given an intensity $i\in[0,b-1]$, the probability $p(I=i)$ is computed as the number of pixel locations $\mathbf{x}$ with $I(\mathbf{x}) = i$ divided by the total number of pixels:

$$p(I=i) = \frac{|\{\mathbf{x}\mid I(\mathbf{x})=i\}|}{|I|} \qquad p(R=r) = \frac{|\{\mathbf{x}\mid R(\mathbf{x})=r\}|}{|R|} \tag{33}$$

The Mutual information MI is then defined as

$$\mathrm{MI}(I,R) = \sum_{i,r}p(I=i,R=r)\log\left(\frac{p(I=i,R=r)}{p(I=i)p(R=r)}\right) \tag{34}$$

where the sum is taken over all possible intensity values $i,r\in[0,b-1]$ in the images I and R. MI can be expressed in terms of the entropy $\mathrm{H}(I)$, $\mathrm{H}(R)$ and $\mathrm{H}(I,R)$ related the distributions $p(I)$, $p(R)$ and $p(I,R)$, respectively. We have:

$$\begin{aligned}\mathrm{H}(I) &= -\sum_i p(I=i)\log p(I=i) & \mathrm{H}(R) &= -\sum_r p(R=r)\log p(R=r)\\ \mathrm{H}(I,R) &= -\sum_{i,r}p(I=i,R=r)\log p(I=i,R=r) & \mathrm{MI}(I,R) &= \mathrm{H}(I)+\mathrm{H}(R)-\mathrm{H}(I,R)\end{aligned} \tag{35}$$

The normalized mutual information (NMI) is defined as follows:

$$\mathrm{NMI}(I,R) = \frac{\mathrm{MI}(I,R)}{\mathrm{H}(I,R)} + 1 = \frac{\mathrm{H}(I) + \mathrm{H}(R)}{\mathrm{H}(I,R)} \tag{36}$$

NMSE (Normalized Mean Squared Error)

For images I and R, the normalized mean squared error is defined as

$$\mathrm{NMSE}(I,R) = \frac{1}{|I| \cdot \sigma_R} \cdot \sum_{\mathbf{x}} (R(\mathbf{x}) - I(\mathbf{x}))^2 \tag{37}$$

where σ_R denotes the corrected sample standard deviation of the intensity value distribution of image R.

PCC (Pearson Correlation Coefficient)

$$\mathrm{PCC}(I,R) = \frac{\sum_{\mathbf{x}} (I(\mathbf{x}) - \mu_I)(R(\mathbf{x}) - \mu_R)}{\sqrt{\sum_{\mathbf{x}} (I(\mathbf{x}) - \mu_I)^2} \sqrt{\sum_{\mathbf{x}} (R(\mathbf{x}) - \mu_R)^2}} \tag{38}$$

where the summations are taken over all pixel locations $\mathbf{x}$ and $R(\mathbf{x})$, $I(\mathbf{x})$ denote the respective intensity values at that location.

PSNR (Peak Signal-to-Noise Ratio)

Please see MSE for the mean squared error.

$$\mathrm{PSNR}(I,R) = 10 \cdot \log_{10}\left(\frac{L^2}{\mathrm{MSE}(I,R)}\right) = 20 \cdot \log_{10}(L) - \log_{10}(\mathrm{MSE}(I,R)) \tag{39}$$

RMSE (Root Mean Squared Error)

$$\mathrm{RMSE}(I,R) = \sqrt{\frac{1}{|I|} \sum_{\mathbf{x}} (R(\mathbf{x}) - I(\mathbf{x}))^2} \tag{40}$$

SSIM (Structural Similarity Index Measure)

The structural similarity index measure (SSIM) combines local image luminance, contrast, and structure. Mean, standard deviation and covariance are calculated locally for each pixel location $\mathbf{x}$ within a d-dimensional Gaussian kernel of size 11 and $\sigma = 1.5$ and are denoted by $\mu_I(\mathbf{x})$, $\sigma_I(\mathbf{x})$, $\mu_R(\mathbf{x})$, $\sigma_R(\mathbf{x})$ and $\sigma_{I,R}(\mathbf{x})$ respectively. For each pixel location $\mathbf{x}$ and its local neighborhood in images I and R, the luminance l, contrast c and structure s are defined as:

$$\begin{aligned}
\mathrm{l}(I,R,\mathbf{x}) &= \left(\frac{2\sigma_I(\mathbf{x})\sigma_R(\mathbf{x}) + C_2}{\sigma_I(\mathbf{x})^2 + \sigma_R(\mathbf{x})^2 + C_2}\right)^{\alpha} \\
\mathrm{c}(I,R,\mathbf{x}) &= \left(\frac{2\mu_I(\mathbf{x})\mu_R(\mathbf{x}) + C_1}{\mu_I(\mathbf{x})^2 + \mu_R(\mathbf{x})^2 + C_1}\right)^{\beta} \\
\mathrm{s}(I,R,\mathbf{x}) &= \left(\frac{\sigma_{I,R}(\mathbf{x}) + C_3}{\sigma_I(\mathbf{x})\sigma_R(\mathbf{x}) + C_3}\right)^{\gamma}
\end{aligned} \tag{41}$$

where C_1, C_2, C_3 are constants to avoid division by arbitrarily small numbers. The local luminance, contrast and structure are then multiplied and averaged for all pixel locations $\mathbf{x}$. Commonly, structure, luminance and contrast terms are weighted equally with $\alpha = \beta = \gamma = 1$. Choosing in addition $C_3 = C_2/2$ yields a simplified formula:

$$\mathrm{SSIM}(I,R) = \frac{1}{|I|} \sum_{\mathbf{x} \in R} \frac{(2\mu_I(\mathbf{x})\mu_R(\mathbf{x}) + C_1) \cdot (2\sigma_{I,R}(\mathbf{x}) + C_2)}{(\mu_I(\mathbf{x})^2 + \mu_R(\mathbf{x})^2 + C_1) + (\sigma_I(\mathbf{x})^2 + \sigma_R(\mathbf{x})^2 + C_2)} \tag{42}$$

The other constants are commonly selected as $C_1 = (0.01 \cdot L)^2$ and $C_2 = (0.03 \cdot L)^2$ with $L = 255$ defined as the data range of the intensity values, in the common case of 8-bit unsigned integers, $L = 255$[6], otherwise $L = \max(I_{\max}, R_{\max}) - \min(I_{\min}, R_{\min})$. See also CW-SSIM and MS-SSIM.

VL (Variance of Laplacian)

The Laplacian of an image I can be calculated by convolution ($*$) with the Laplacian filter:

$$\mathrm{Laplace}(I) = I * \begin{pmatrix} 0 & 1 & 0 \\ 1 & -4 & 1 \\ 0 & 1 & 0 \end{pmatrix} \tag{43}$$

Then, the variance is calculated:

$$\mathrm{VL}(I) = \frac{1}{|I|} \sum_{\mathbf{x} \in I} \left(\mathrm{Laplace}(I)(\mathbf{x}) - \mu_{\mathrm{Laplace}(I)}\right)^2 \tag{44}$$

A.4 Calculation of Distortions

For each type of distortion, the range of parameters is given for strengths $s = 1$ to $s = 5$. All other strengths are interpolated linearly between the given values. In addition to the notation in Sec. A.1., the distorted image is denoted as $\tilde{I}$. Let $\mathbf{s} = (w, h)$ be the image size vector of I. The minimum and maximum intensity of I are denoted by $I_{\min}$, $I_{\max}$. Inverted transformations, that can restore a transformed image, are denoted by $^{-1}$, e.g. Minmax^{-1}, FFT^{-1} or shift^{-1}. Distortions are ordered alphabetically.

Bias Field, $c \in [0.5, 10]$
An artificial bias field is created from the polynomial function P_3 and applied to the image:

$$P_3(x_1, x_2) = 10x_1^2(x_1 - 1) \cdot (x_2 - 0.5)x_2(x_2 - 1) \quad (45)$$

$$\tilde{I} = I \cdot e^{c \cdot P_3} \quad (46)$$

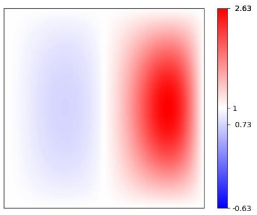

$P_3(x_1, x_2)$

Elastic Deform, $n \in [18, 11], d \in [0.03, 0.1]$
A grid with n^2 points is created, and points are displaced by a displacement vector sampled from $\mathcal{N}(\mu = \mathbf{0}; \sigma = d \cdot \frac{\mathbf{s}}{n})$. The image is interpolated along the grid.

Gamma Low / Gamma High, $\log(\gamma) \in [-0.01, -0.916]$ / $\log(\gamma) \in [0.095, 0.916]$

$$\tilde{I} = \text{Minmax}^{-1}(\text{Minmax}(I)^{\gamma}) \quad (47)$$

Gaussian Blur, $\sigma \in [0.2, 1.3]$
The image is convoluted with a Gaussian kernel G_σ:

$$\tilde{I} = \text{Conv}(G_\sigma, I) \quad (48)$$

Gaussian Noise, $\sigma \in [0.005, 0.05]$

$$\tilde{I} = I + \mathcal{N}(\mu = 0; \sigma) \quad (49)$$

Ghosting, $i \in [0.05, 0.4]$
The fast Fourier transform (FFT) transforms the image to the frequency domain. The shift operator then moves the center of the transformed image to 0. The spectrum is scaled (distorted) at every second pixel (to generate two ghosts) along the first axis, then the image is restored for all pixels with $x_1 = w/2$.

$$I' = \text{shift}(\text{FFT}(I)) \quad (50)$$

$$I''(x_1, x_2) = I' \cdot i, \text{ for } x_1 \,\%\, 2 = 0 \quad (51)$$

$$I''(x_1, x_2) = I', \text{ for } x_1 = w/2 \quad (52)$$

$$\tilde{I} = \text{shift}^{-1}(\text{FFT}^{-1}(I'')) \quad (53)$$

Replace Artifact, $f \in [0.1, 1.0]$
Copy and mirror a fraction of the upper half of the image to the lower half:

$$\tilde{I}(x_1, x_2) = \begin{cases} I(x_1, x_2) & \text{, if } x_2 \leq \frac{h}{2} \\ I(x_1, h - x_2) & \text{, if } x_2 > \frac{h \cdot (1+f)}{2} \end{cases} \quad (54)$$

Shift Intensity, $f \in [0.05, 0.25]$

$$\tilde{I} = I + f \cdot (I_{\max} - I_{\min}) \quad (55)$$

Stripe Artifact, $i \in [0.05, 0.5]$
The fast Fourier transform (FFT) transforms the image to the frequency domain. The shift operator then moves the center of the transformed image to 0. The spectrum is scaled (distorted) at a single pixel $(0.3 \cdot \cos(0), 0.3 \cdot \sin(0)$. Then the image is transformed back to the spatial domain again.

$$\begin{aligned} I' &= \text{FFT}(\text{shift}(I)) \\ I''(x_1, x_2) &= i \cdot I_{\max}, \text{ for } x_1 = 0.3 \cdot \cos(0),\ x_2 = 0.3 \cdot \sin(0) \\ \tilde{I} &= \text{FFT}^{-1}(\text{shift}^{-1}(I'')) \end{aligned} \quad (56)$$

Translation, $f \in [0.01, 0.2]$

$$\tilde{I}(x_1, x_2) = I(f \cdot w + x_1, f \cdot h + x_2) \quad (57)$$

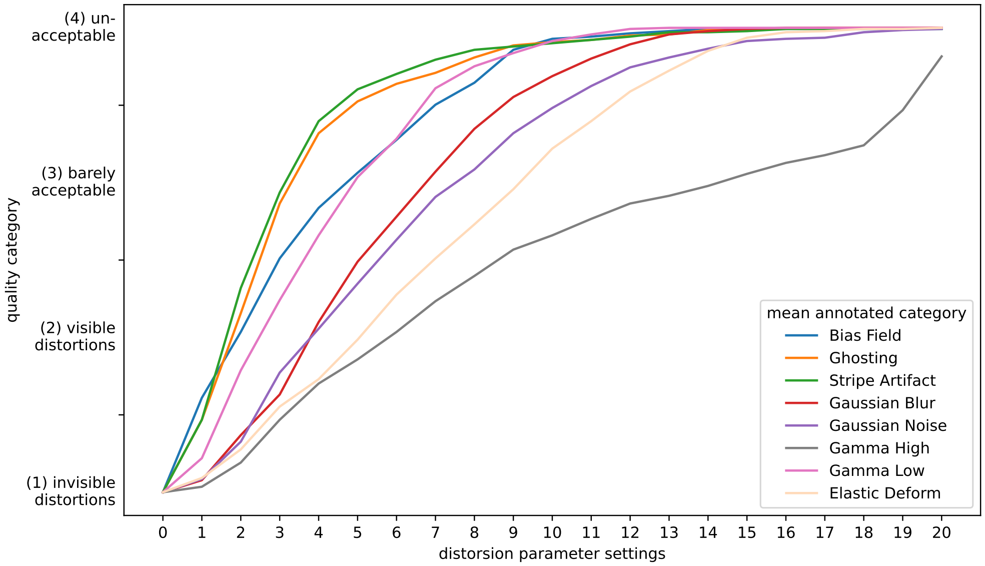

Figure S.1. Mean category assigned by the six readers for 20 initial parameter settings for each distortion.

A.5 Reader study for distortion parameterization

For finding reasonable and comparable distortion parameter ranges, a reader study was conducted. Six researchers with at least three years experience in the field of medical image anaylsis were asked to partition a sequence of images with increasing distortion parameter settings into four categories:

1. distortions not visible compared to the reference image, perfect quality
2. visible distortions, but irrelevant difference to the reference, very good quality
3. barely acceptable distortions, could be useful for certain diagnostic questions, poor quality
4. excessive distortions, images cannot be used for any diagnostic question

All distorted image versions belonging to one reference image were shown at a time, in a scrollable table of nine distortions and 20 distortion parameter settings. For 20 reference images (20 T1-weighted images of the BraTS dataset, 10 of them contrast enhanced), the proposed categorization was evaluated. Additionally, comments of the readers were possible, in case all presented images were perceived outside of category 1 or 4.
In the reader study, all images were normalized to an 8-bit range by Minmax normalization. The distortions were applied to this range and then clipped to the (0, 255) range. This was done in order to visualize and overview the entire intensity value range of the reference and distorted images. The median of the strongest distortion parameter setting, that was assigned to category 1, s_{min}, was later used to find a parameter setting for strength = 1. The median of the weakest distortion parameter setting, that was assigned to category 4, s_{max}, was later used to find a parameter setting for strength 5.
As a result from the reader study (see Fig. S.1), including collected comments, the initial parameter ranges for Gamma High and Ghosting were extended, in order to better cover the desired quality categories. For the final experiments, further minor adaptions were made to the distortions to guarantee non-varying stripes, and an equally darkening and brightening bias field. In most cases, distortion intensity parameters were additionally multiplied with the image intensity value range, allowing to apply distortions to the original full MR image intensity range. This allows our analysis to evaluate reference and distorted images with their full intensity range without information loss. Furthermore, the effect of different normalization methods can be investigated.
The parameterization of the investigated distortions was initialized from results of the reader study on images reduced to 8-bit. The transfer of these parameters to images with the original MR intensity range was estimated by the authors. Directly deriving the parameters from the reader study would impose reducing the intensity value ranges of the images being inspected to a visible range. How this can be solved is ongoing research and has been questioned, also in [7].

B Supplementary Figures

B.1 Sensitivity to Strength of Spatial distortions

Metric scores for different distortion strengths of Translation and Elastic Deform were compared. All metrics scores shown here were assessed on images without normalization, except LPIPS, DISTS and DSC. *: For LPIPS Minmax normalization to [-1, 1] was used, for DISTS Minmax normalization to [0, 1] was applied and DSC was assessed after Zscore normalization and segmentation.

Distortions (rows) / metrics (columns)	s	SSIM↑	MS-SSIM↑	CW-SSIM↑	PSNR↑	MSE↓	NMSE↓	MAE↓	LPIPS*↓	DISTS*↓	NMI↑	PCC↑	DSC*↑
Reference	0	1.00	1.00	1.00	∞	0.00	0.00	0.00	0.00	0.00	2.00	1.00	1.00
Translation	1	0.83	0.87	0.98	26.39	143.10	$2.13 \cdot 10^5$	186.02	0.07	0.08	1.27	0.92	0.83
Elastic Deform	5	0.88	0.92	0.91	28.24	105.88	$1.30 \cdot 10^5$	116.73	0.06	0.11	1.32	0.95	0.86

B.2 Examples of Distorted Images

In this section we provide more examples of distorted images for all distortions and all strengths for the reader to get an impression about the distortion strengths on different images. The images are visualized with differently intensity ranges, specified in the captions.

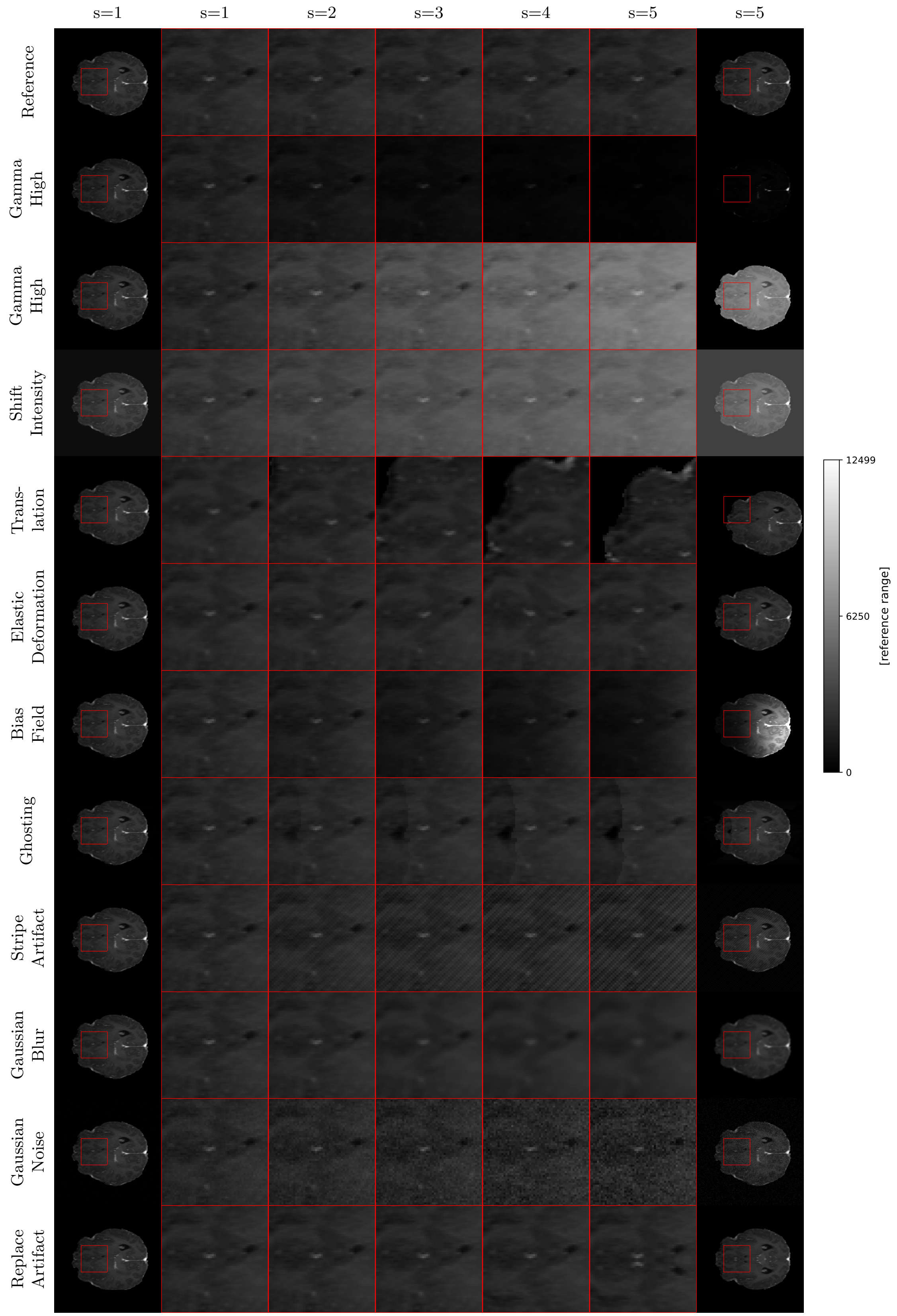

Figure S.2. Distorted versions of BraTS-GLI-00005-000-t1c, visualized with the intensity range of the reference image.

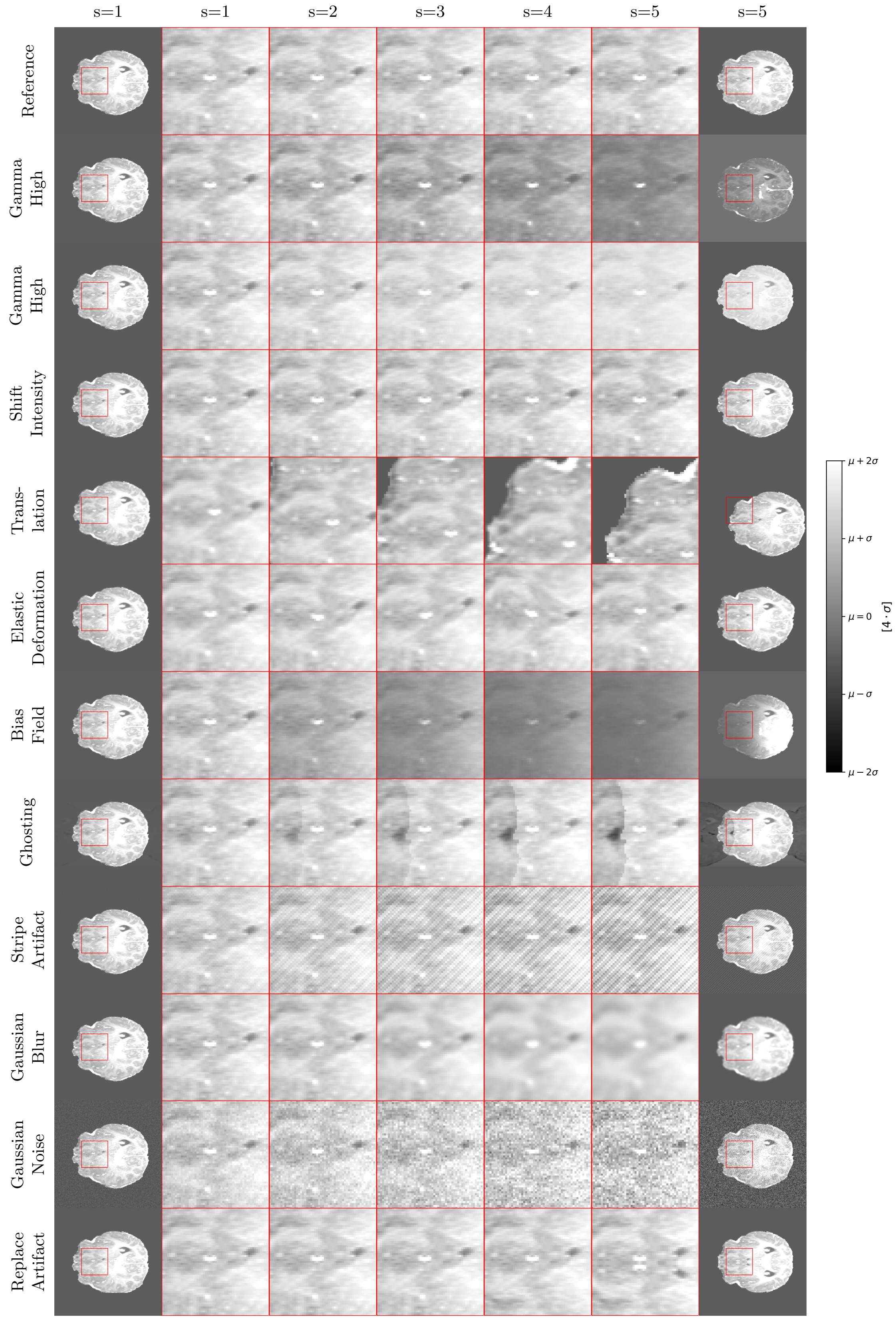

Figure S.3. Distorted versions of BraTS-GLI-00000-005-t1c, visualized with an intensity range of four standard deviations around the mean of each image individually.

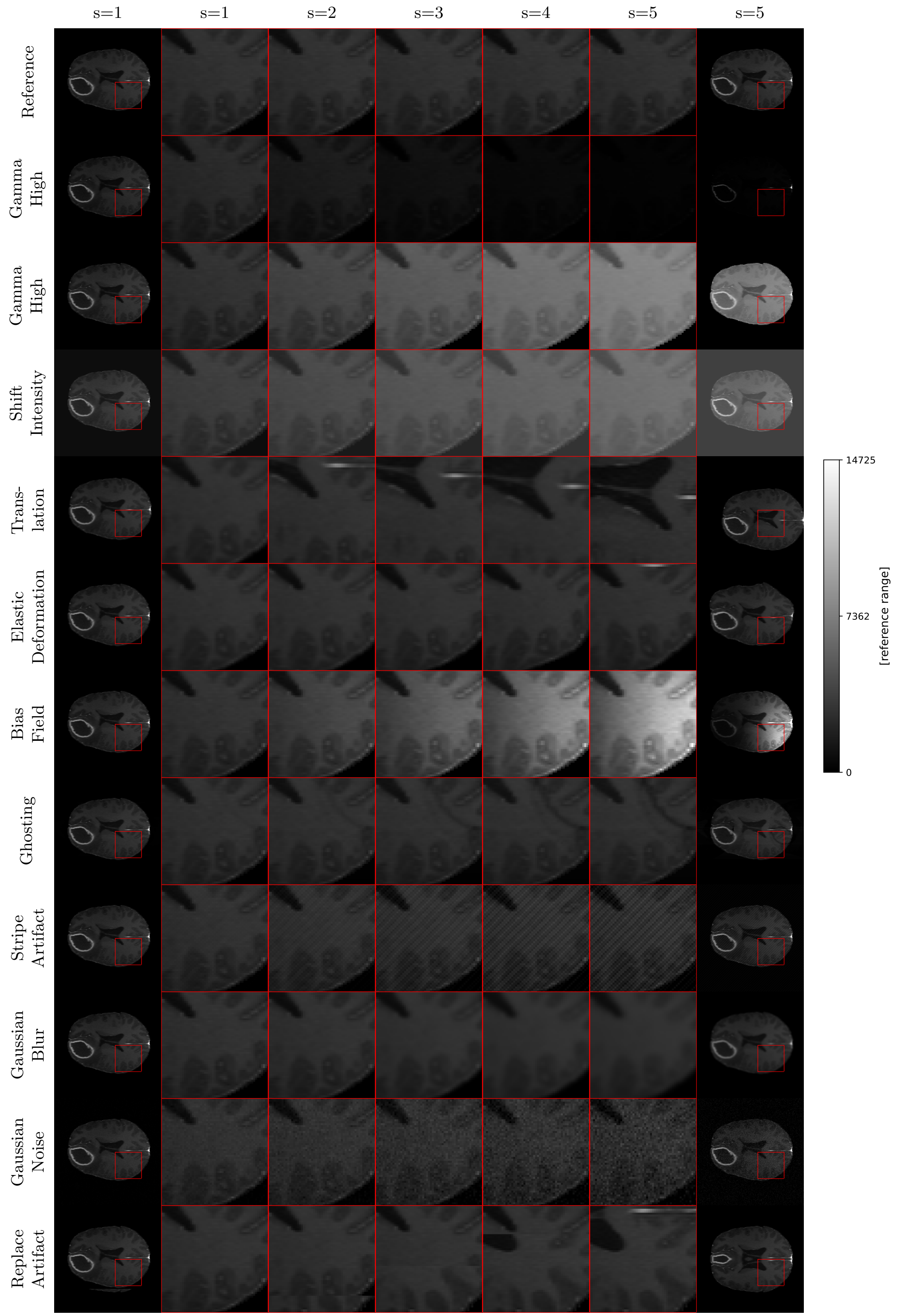

Figure S.4. Distorted versions of BraTS-GLI-00006-000-t1c, visualized with the intensity range of the reference image.

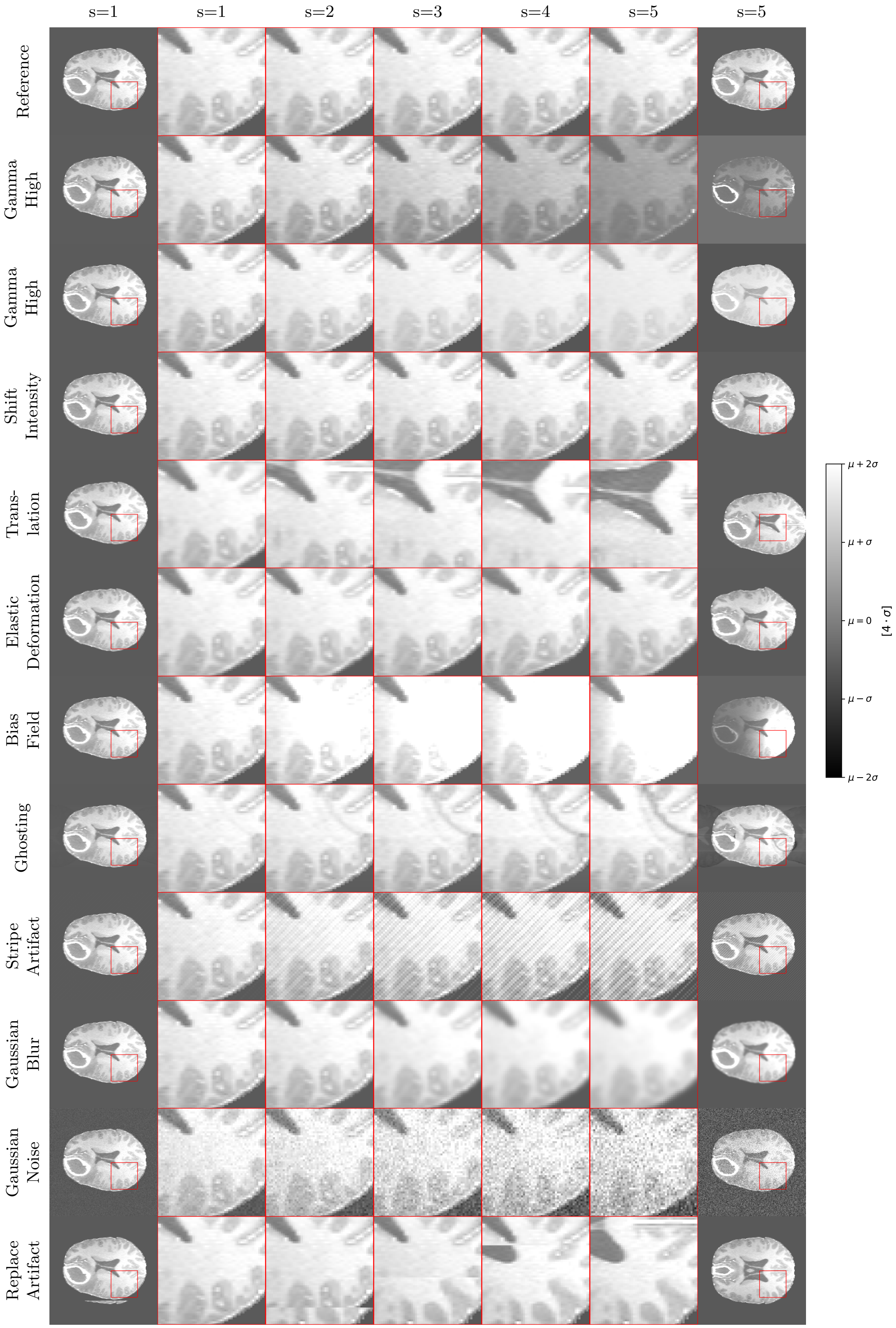

Figure S.5. Distorted versions of BraTS-GLI-00006-000-t1c, visualized with an intensity range of four standard deviations around the mean of each image individually.

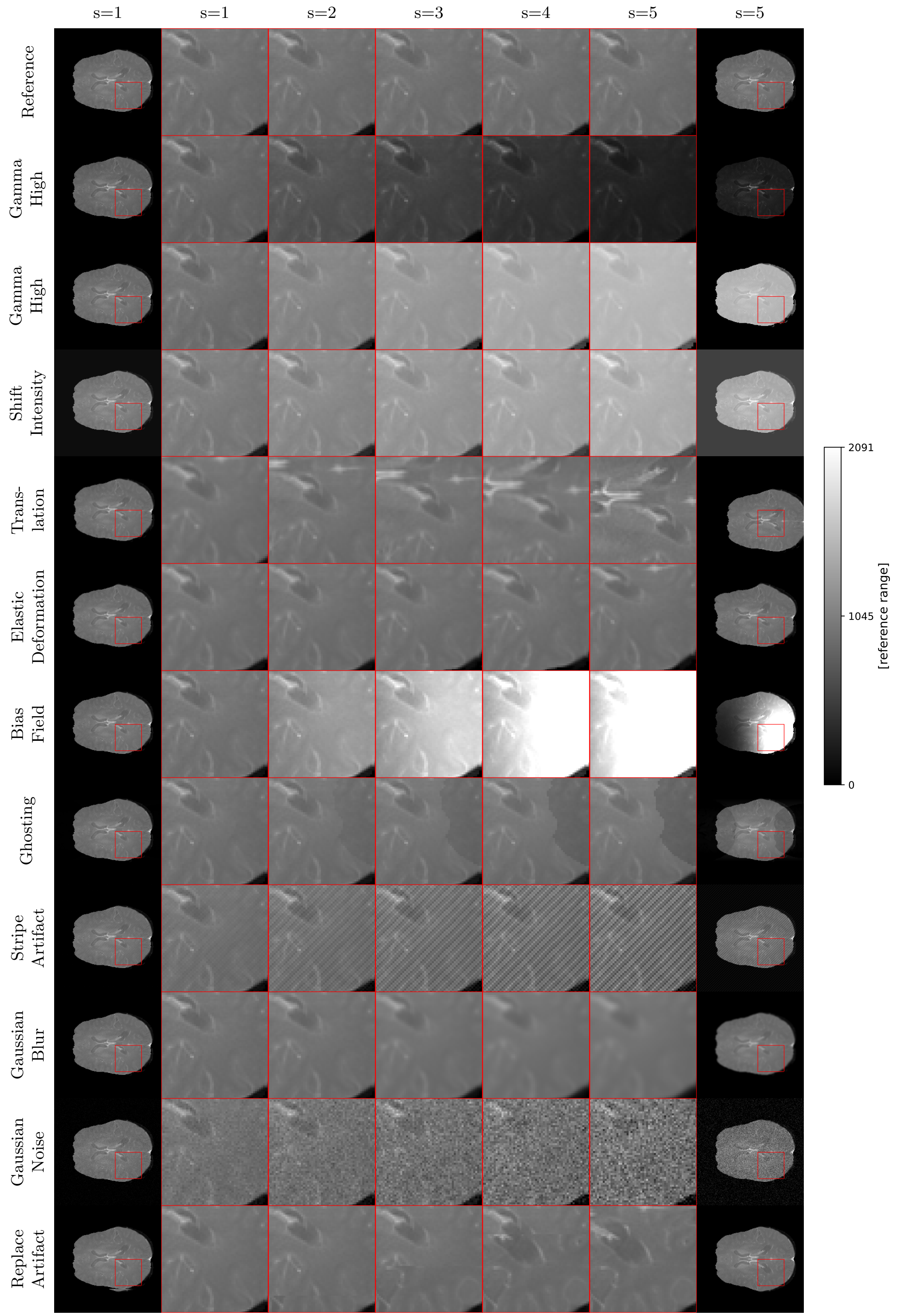

Figure S.6. Distorted versions of BraTS-GLI-00014-001-t1c, visualized with the intensity range of the reference image.

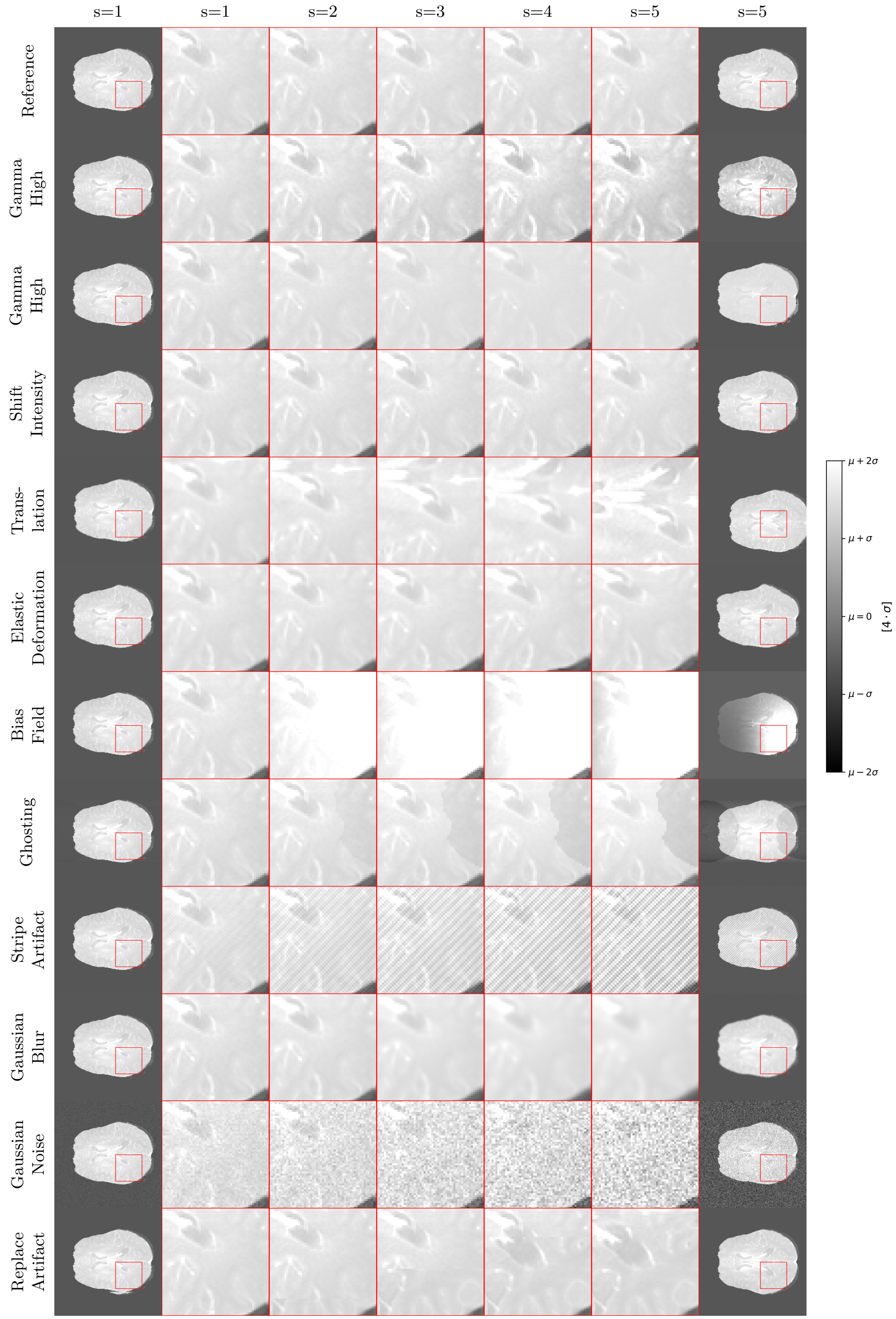

Figure S.7. Distorted versions of BraTS-GLI-00014-001-t1c, visualized with an intensity range of four standard deviations around the mean of each image individually.

B.3 Evaluation Plots for All Reference Metrics and Normalization Methods

In the following figures we present six plots for each combination of reference metric, one for each normalization method, including w/o normalization. For each distortion and strength the median of all metric scores across all 100 cases is plotted, such that an increasing or decreasing trend is usually observed along the distortion strengths.

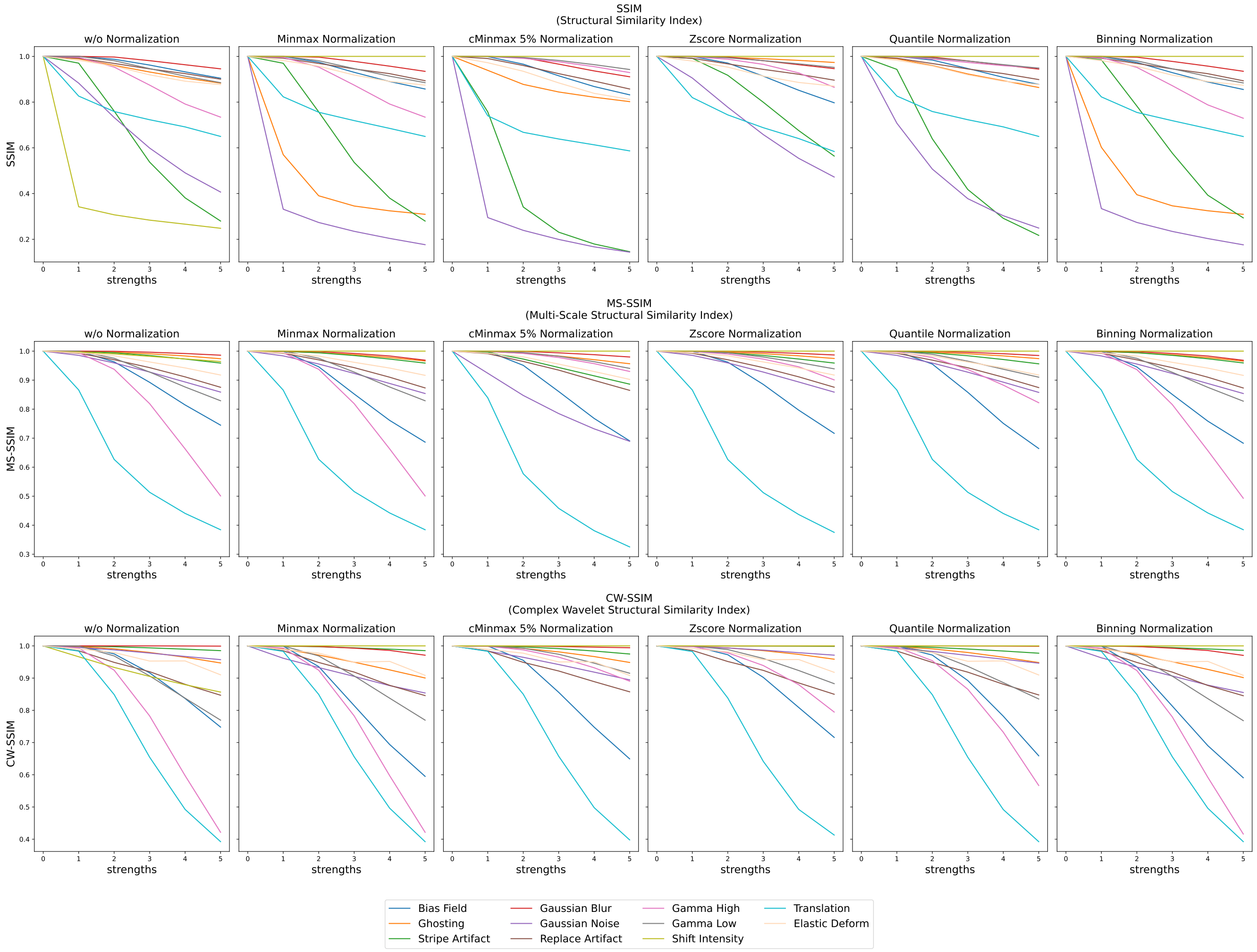

Figure S.8. Median scores of SSIM-based reference metrics SSIM (top), MS-SSIM (middle), and CW-SSIM (bottom) across 100 images, distorted with increasing strengths (0: reference, 1: hardly/not visibly distorted, 5: strongly distorted), grouped by kinds of distortions in different colors.

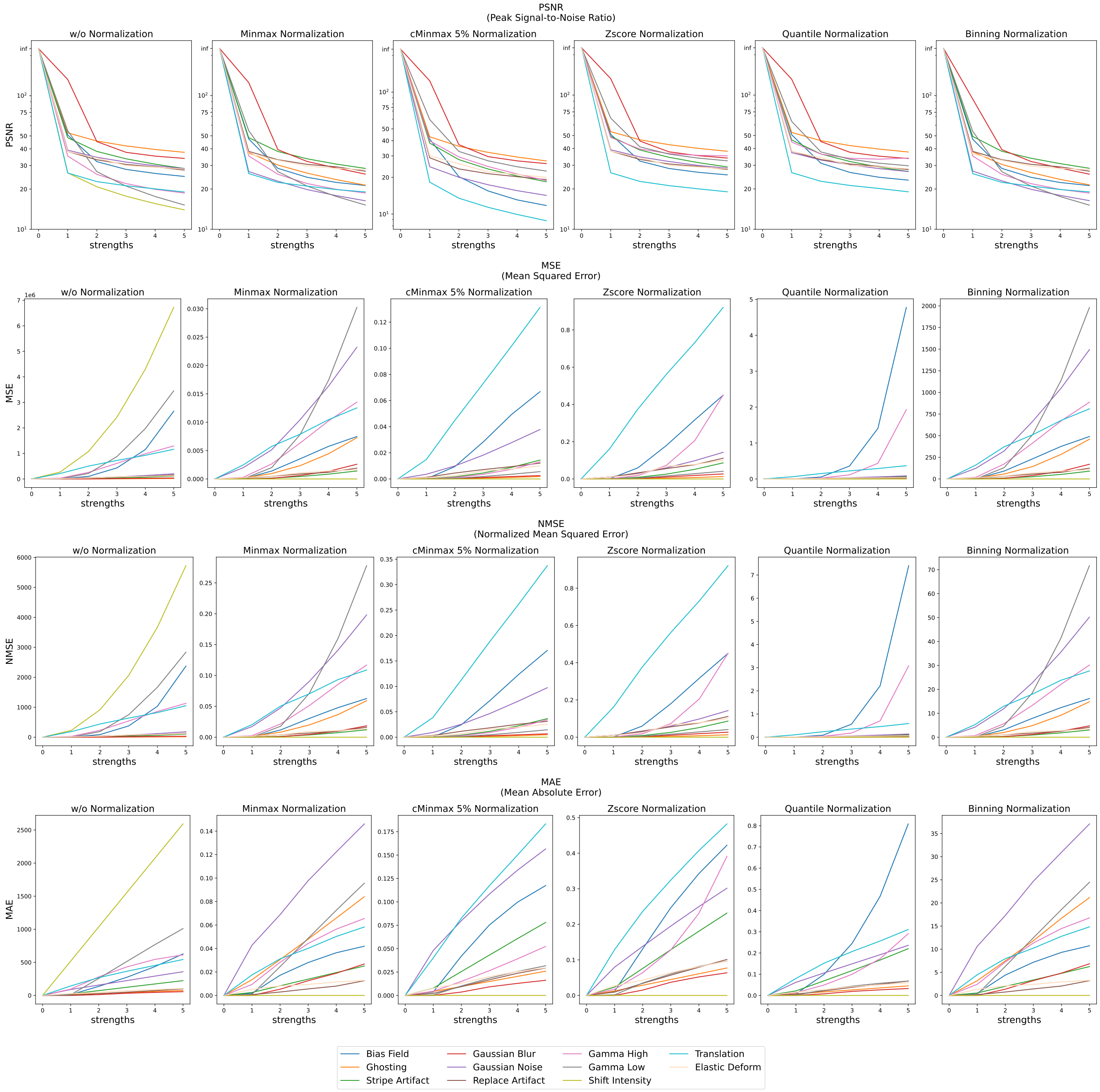

Figure S.9. Median scores of error-based reference metrics PSNR (top), MSE (second row), NMSE (third row) and MAE (bottom) across 100 images, distorted with increasing strengths (0: reference, 1: hardly/not visibly distorted, 5: strongly distorted), grouped by kinds of distortions in different colors.

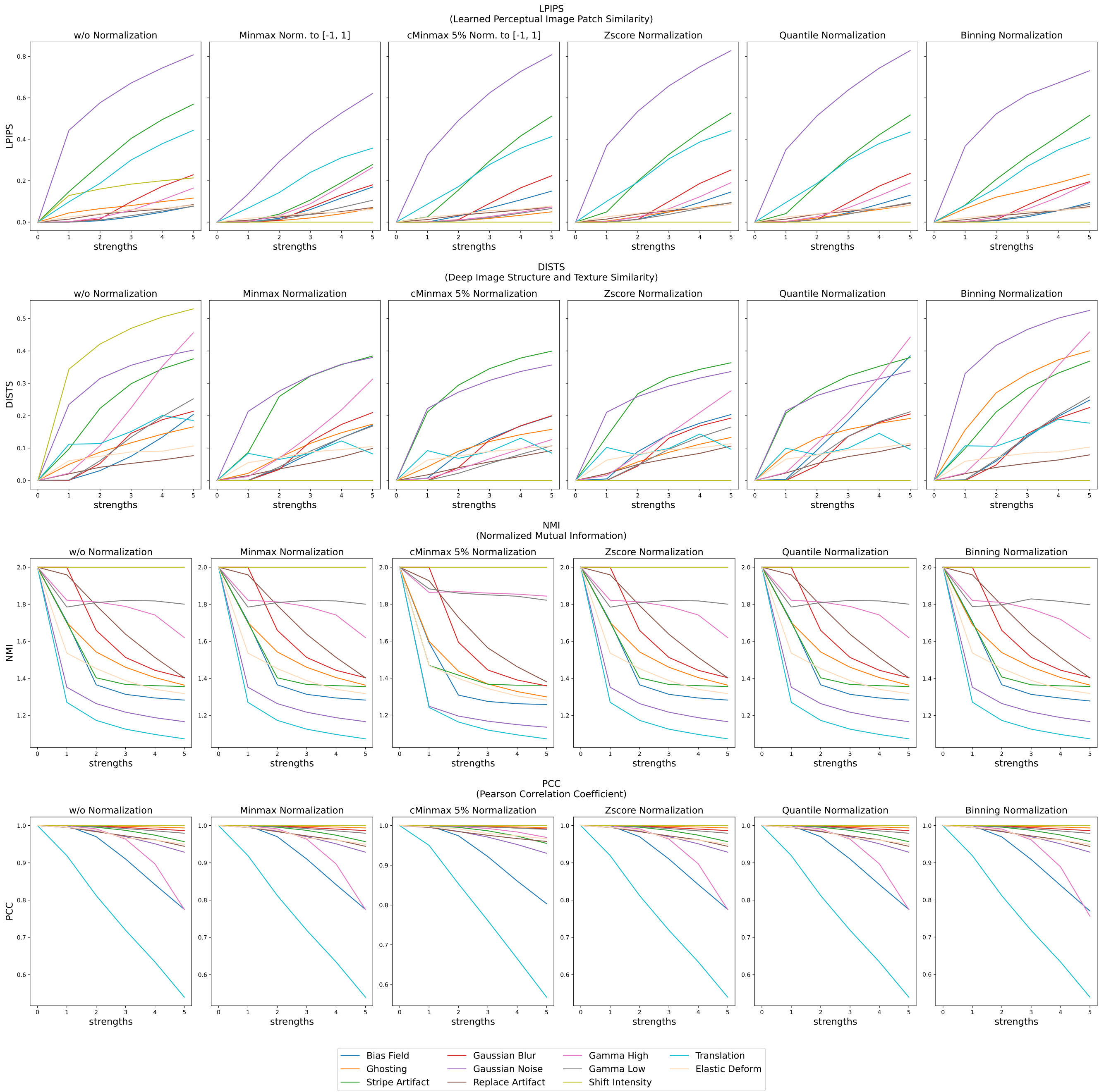

Figure S.10. Median scores of the learned reference metrics LPIPS (top) and DISTS (second row), and statistical dependency reference metrics NMI (third row) and PCC (bottom) across 100 images, distorted with increasing strengths (0: reference, 1: hardly/not visibly distorted, 5: strongly distorted), grouped by kinds of distortions in different colors. LPIPS and DISTS require normalized images with an intensity range around 0, therefore, analysis of these metrics was not performed without normalization.

B.4 Evaluation Plots for All Non-Reference Metrics and Normalization Methods

In the following figures we present six plots for each combination of reference metric, one for each normalization method, including w/o normalization. For each distortion and strength the median of all metric scores across all 100 cases is plotted, such that an increasing or decreasing trend is usually observed along the distortion strengths. Most non-reference metrics were designed to be applied to 8-bit integer images, which is the case after Binning normalization.

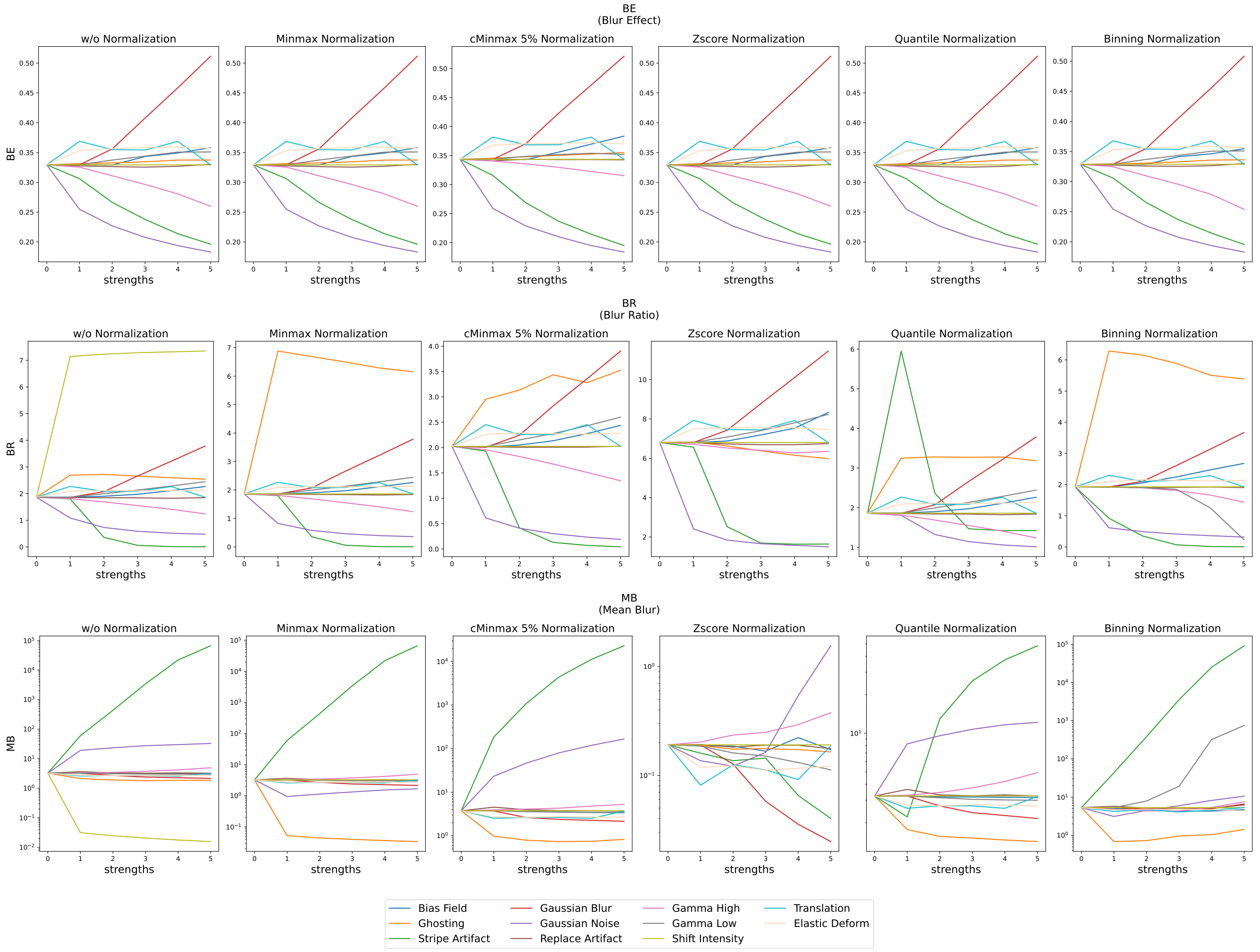

Figure S.11. Median scores of non-reference blurriness metrics BE (top), BR (middle) and MB (bottom) across 100 images, distorted with increasing strengths (0: reference, 1: hardly/not visibly distorted, 5: strongly distorted), grouped by kinds of distortions in different colors.

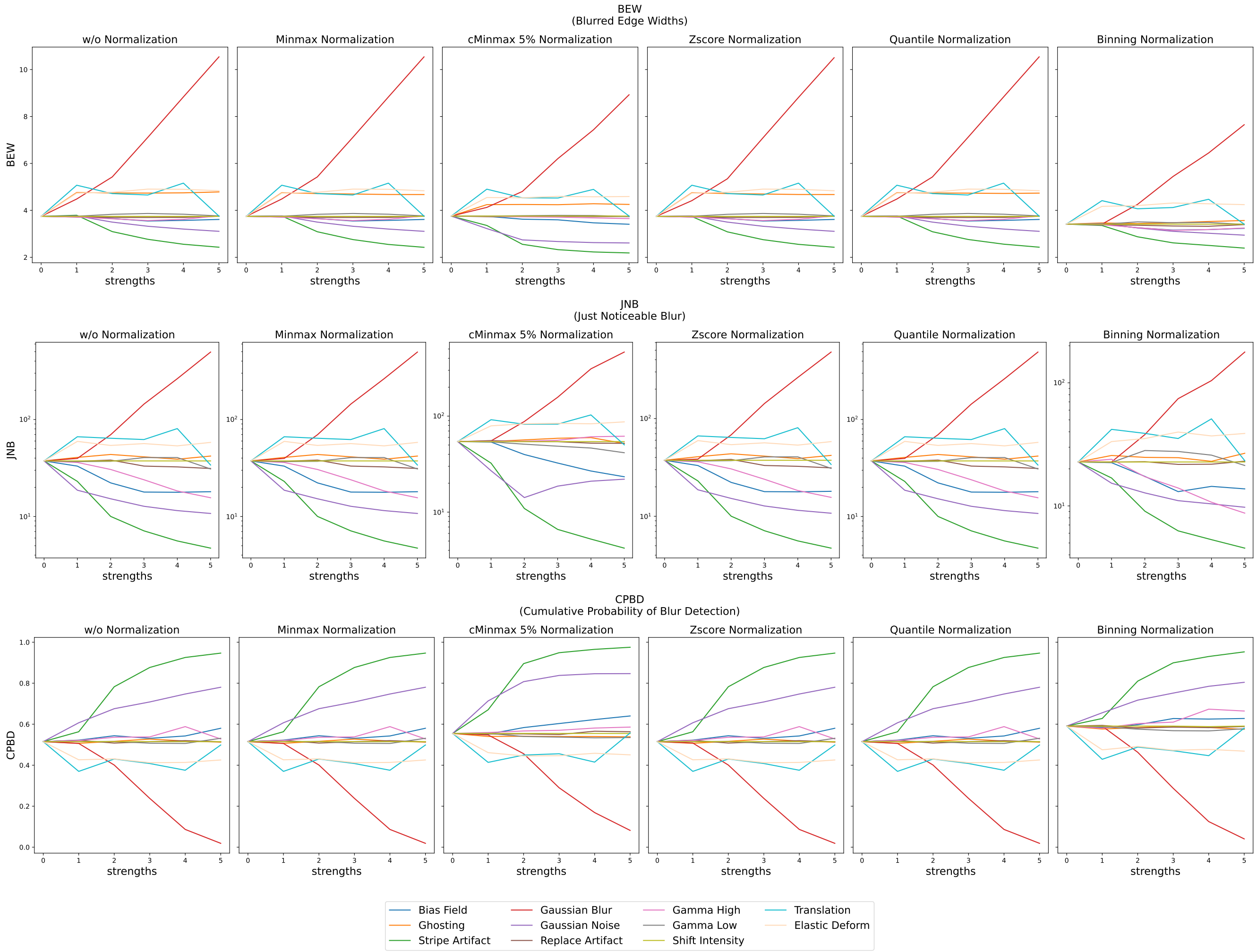

Figure S.12. Median scores of non-reference blurriness metrics BEW (top), JNB (middle) and CPBD (bottom) across 100 images, distorted with increasing strengths (0: reference, 1: hardly/not visibly distorted, 5: strongly distorted), grouped by kinds of distortions in different colors.

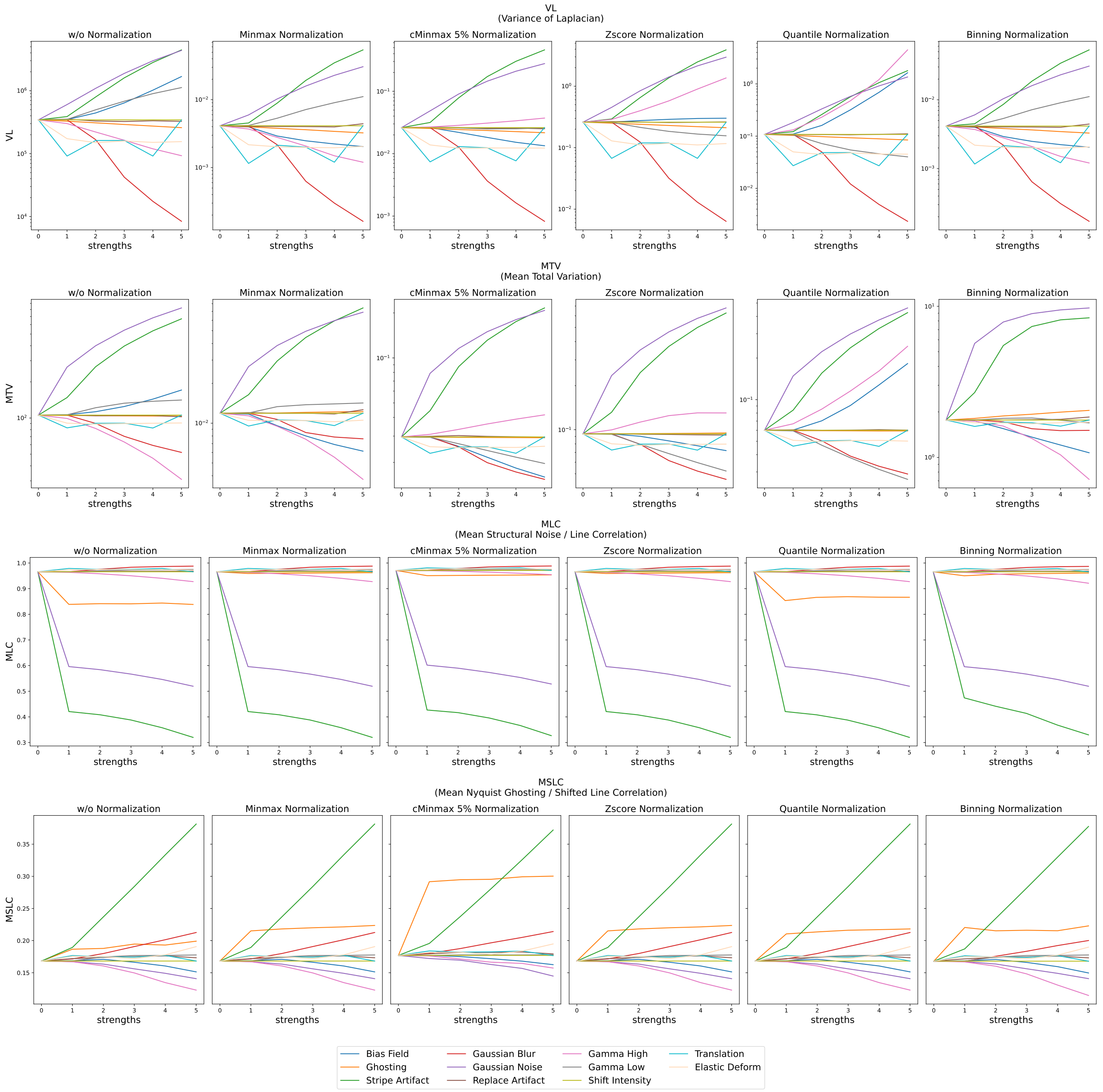

Figure S.13. Median scores of further non-reference blurriness, noisiness and MR acquisition quality metrics VP (top), MTV (second row), MLC (third row), and MSLC (bottom) metric across 100 images, distorted with increasing strengths (0: reference, 1: hardly/not visibly distorted, 5: strongly distorted), grouped by kinds of distortions in different colors.

,

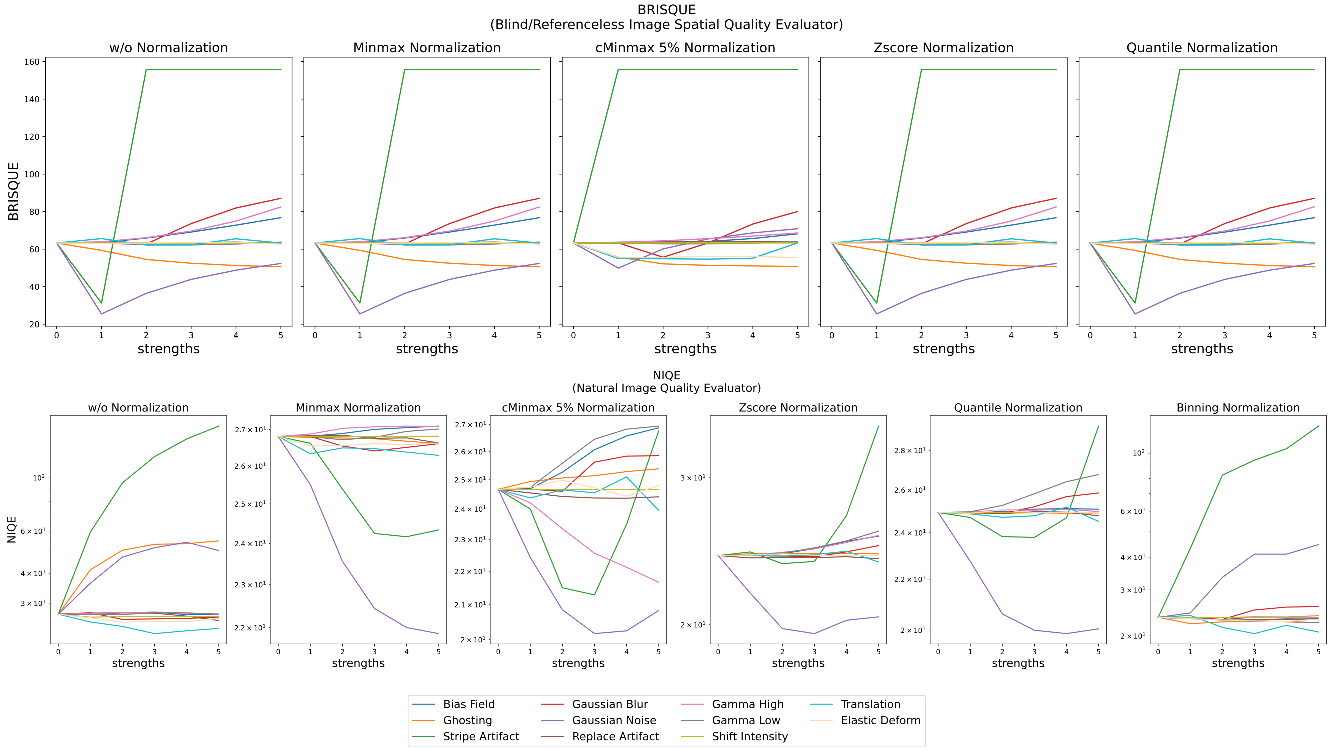

Figure S.14. Median scores of the learned non-reference quality metrics BRISQUE (top), and NIQE (bottom) metric across 100 images, distorted with increasing strengths (0: reference, 1: hardly/not visibly distorted, 5: strongly distorted), grouped by kinds of distortions in different colors.

B.5 Evaluation Plots for Downstream Task Metric

In the following figures we present six plots for each combination of reference metric, one for each normalization method, inlcuding w/o normalization. For each distortion and strength the median of all metric scores across all 100 cases is plotted, such that an increasing or decreasing trend is usually observed along the distortion strengths.

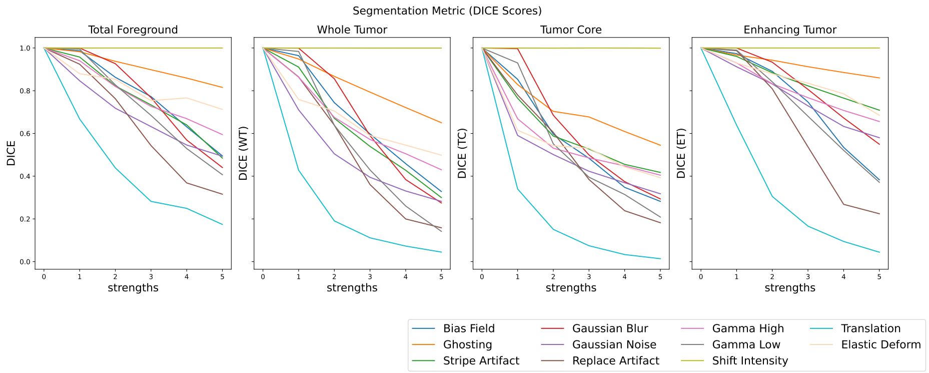

Figure S.15. Mean scores of the DSC segmentation metric across 100 segmentation pairs derived from a reference a distorted image with increasing strengths applied (0: reference, 1: hardly/not visibly distorted, 5: strongly distorted). The mean scores are grouped by kinds of distortions in different colors. The "Total Foreground" class includes the three disjoint classes "Whole Tumor" (mainly tumor surrounding edema), "Tumor Core" (mainly necrotic areas) and "Enhancing Tumor" (vital tumor cells, taking up contrast media).

References

1. van der Walt, S. *et al.* scikit-image: image processing in Python. *PeerJ* **2**, e453, DOI: 10.7717/peerj.453 (2014).
2. Canny, J. A computational approach to edge detection. *IEEE Transactions on Pattern Analysis Mach. Intell.* **PAMI-8**, 679–698, DOI: 10.1109/TPAMI.1986.4767851 (1986).
3. Mittal, A., Moorthy, A. K. & Bovik, A. C. No-reference image quality assessment in the spatial domain. *IEEE Transactions on Image Process.* **21**, 4695–4708, DOI: 10.1109/TIP.2012.2214050 (2012).
4. Ding, K. Iqa optimization. https://github.com/dingkeyan93/IQA-optimization/ (2020).
5. Zhang, R., Isola, P., Efros, A. A., Shechtman, E. & Wang, O. The unreasonable effectiveness of deep features as a perceptual metric. *CoRR* **ayn/1801.03924** (2018). 1801.03924.
6. Wang, Z., Bovik, A. C., Sheikh, H. R. & Simoncelli, E. P. Image quality assessment: from error visibility to structural similarity. *IEEE Transactions on Image Process.* **13**, 600–12 (2004).
7. Kastryulin, S., Zakirov, J., Pezzotti, N. & Dylov, D. V. Image quality assessment for magnetic resonance imaging. *IEEE Access* **11**, 14154–14168, DOI: 10.1109/ACCESS.2023.3243466 (2023).
